# Health Tract, No. 62

**Published:** 1862-02

**Authors:** 


### HEALTH TRACT, No. 62.

### HEADACHE

Is generally not a disease in the head itself, but a sign or symptom that something is wrong in some other part of the body. In almost every case it is accompanied by cold feet, costiveness, disordered stomach, or a derangement of the nervous system in general—this last induced by over-mental exercise or some local irritation in a distant part of the body. In all these cases, an application to the head itself is only palliative, eradicates nothing, cures nothing. If the feet are cold they must be made permanently dry and warm, thus drawing the excess of blood away from the head. If the bowels, are costive they must be made to act once every twenty-four hours, freely and habitually, without the use of any medicine. If headache comes on at regular times after eating, then indigestion is the cause, and such food should be used, both in quantity and quality, as will not be followed by this symptom. But if the feet are habitually warm and comfortable, if the bowels act once regularly every day, and if it is clear that the headache is not connected with the eating, then its cause must be found in some part of the system remote from the head itself, and it is safest and best to take competent medical advice, if trouble, anxiety, or over-mental exertion is not the palpable origin of the ailment. In most cases, very severe headaches will disappear within twenty-four hours, by giving the scalp, face, and whole body a most thorough cleansing with soap, warm water, and a rough scrubbing-rag; taking nothing but cold water and some kind of soup into which has been broken the crust of cold or toasted bread, and some out-door activities for several hours in the forenoon and afternoon also. Headaches in children should always be promptly attended to, as they indicate the approach of serious diseases, as scarlet fever, small pox, measles, and other grave skin affections. Bilious headache is caused by the liver abstracting too much or too little bile from the blood, giving pain in the shoulder, sick stomach, loss of appetite, depression, pain in forehead. Hysteric headache gives pain in a small spot over the eye-brow, as if a nail or wedge were driven in. Pain on the top of the skull often arises from exhaustion or debility. A pain over the brow, coming on at a regular hour daily, not lasting long, is in the nature of fever and ague, and sometimes feels as if the skull was opening and shutting. The *Megrims* are of this nature, the pain beginning at the inner corner of the eye, making it tender and red, sometimes affecting half the head, perfectly disappearing and returning at irregular intervals. Sometimes hereditary. A dull aching or rather soreness, especially on pressure, at the back of the head, brow, or temples, is rheumatic headache, caused by uncovering the head while perspiring; cured by light diet, dry feet, warm clothing, free bowels, and moderate out-door exercise in clear, dry weather.

